# Vasodilatory Effect of *Phellinus linteus* Extract in Rat Mesenteric Arteries

**DOI:** 10.3390/molecules25143160

**Published:** 2020-07-10

**Authors:** Youngin Kwon, Chae Eun Haam, Seonhee Byeon, Soo Jung Choi, Dong-Hoon Shin, Soo-Kyoung Choi, Young-Ho Lee

**Affiliations:** 1 Department of Physiology, College of Medicine, Brain Korea 21 PLUS Project for Medical Sciences, Yonsei University, 50 Yeonseiro, Seodaemun-gu, Seoul 03722, Korea; youngin0618@yonsei.ac.kr (Y.K.); firststep22@naver.com (C.E.H.); seonhee89@yuhs.ac (S.B.); 2 Department of Food and Biotechnology, Korea University, 145 Anam-ro, Seongbuk-gu, Seoul 02841, Korea; sjchoi@korea.ac.kr (S.J.C.); dhshin@korea.ac.kr (D.-H.S.)

**Keywords:** *Phellinus linteus*, K_ca_ channels, vasodilation, relaxation, mesenteric artery

## Abstract

*Phellinus linteus* is a well-known medicinal mushroom that is widely used in Asian countries. In several experimental models, *Phellinus linteus* extracts were reported to have various biological effects, including anti-inflammatory, anti-cancer, hepatoprotective, anti-diabetic, neuroprotective, and anti-angiogenic activity. In the present study, several bioactive compounds, including palmitic acid ethyl ester and linoleic acid, were identified in *Phellinus linteus*. The intermediate-conductance calcium-activated potassium channel (IK_Ca_) plays an important role in the regulation of the vascular smooth muscle cells’ (VSMCs) contraction and relaxation. The activation of the IK_Ca_ channel causes the hyperpolarization and relaxation of VSMCs. To examine whether *Phellinus linteus* extract causes vasodilation in the mesenteric arteries of rats, we measured the isometric tension using a wire myograph. After the arteries were pre-contracted with U46619 (a thromboxane analogue, 1 µM), *Phellinus linteus* extract was administered. The *Phellinus linteus* extract induced vasodilation in a dose-dependent manner, which was independent of the endothelium. To further investigate the mechanism, we used the non-selective K^+^ channel blocker tetraethylammonium (TEA). TEA significantly abolished *Phellinus linteus* extract-induced vasodilation. Thus, we tested three different types of K^+^ channel blockers: iberiotoxin (BK_ca_ channel blocker), apamin (SK_ca_ channel blocker), and charybdotoxin (IK_ca_ channel blocker). Charybdotoxin significantly inhibited *Phellinus linteus* extract-induced relaxation, while there was no effect from apamin and iberiotoxin. Membrane potential was measured using the voltage-sensitive dye bis-(1,3-dibutylbarbituric acid)-trimethine oxonol (DiBAC_4_(3)) in the primary isolated vascular smooth muscle cells (VSMCs). We found that the *Phellinus linteus* extract induced hyperpolarization of VSMCs, which is associated with a reduced phosphorylation level of 20 KDa myosin light chain (MLC_20_).

## 1. Introduction

Resistance arteries are blood vessels that have a small diameter in the microcirculation that contributes to regulation of blood pressure and the distribution of cardiac output within the tissues and organs to meet their physiological demands [1]. The arterial wall consists of a single layer of endothelial cells, vascular smooth muscle cells, elastic fibers, and other extracellular matrix elements. Vascular smooth muscle cells (VSMCs) play significant roles in the functioning of arteries [2]. VSMCs cause the contraction and relaxation of the vascular wall and hence contribute to the regulation of blood flow.

Smooth muscle cells of resistance arteries express various ion channels, including calcium-activated potassium channels (K_ca_ channels) [3]. The K_ca_ channels are a group of abundant K^+^ channels that are activated by increase in intracellular Ca^2+^ concentration, including the small-conductance calcium-activated potassium channel (SK_ca_), the intermediate-conductance calcium-activated potassium channel (IK_ca_), and large-conductance calcium-activated potassium (BK_ca_) channels. The activation of K_ca_ channels enables K^+^ efflux, resulting in hyperpolarization and causing vasodilation. Thus, K_ca_ channel activity is one of the major determinants for vascular tone [4].

*Phellinus linteus* is a well-known medicinal mushroom that is widely used in Korea, Japan, China, and other Asian countries [5]. In several experimental models, it has been reported that *Phellinus linteus* extract contains various phenolic compounds that exert various biological effects, including anti-inflammatory [6,7], anti-cancer [8,9], hepatoprotective [10,11], anti-diabetic [12,13], and neuroprotective [14,15] effects. Recently, it has been shown that *Phellinus linteus* exhibited anti-angiogenic activity in mice [16,17]. Although the biological activities of *Phellinus linteus* extract have been widely reported, the vascular effect of *Phellinus linteus* extract has not been investigated. Thus, in the present study, we investigated whether *Phellinus linteus* extract has effects on the mesenteric resistance arteries of rats, and if so, what the underlying mechanisms were.

## 2. Results

### 2.1. Gas Chromatograms of the Compounds in Phellinus linteus Extract

**Figure 1:**
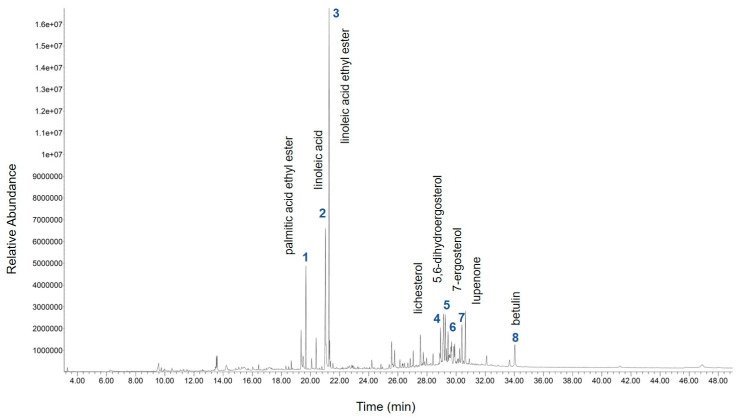
Gas chromatogram of the compounds in *Phellinus linteus* extract.

The gas chromatogram of the compounds identified in the sample of *Phellinus linteus* extract is demonstrated in Figure 1. The identities of eight compounds were determined, along with their retention time (Table 1). The compounds identified based on the gas chromatography–mass spectrometry (GC/MS) analysis include palmitic acid ethyl ester, linoleic acid, linoleic acid ethyl ester, lichesterol, 5,6-dihydroergosterol, 7-ergostenol, lupenone, and betulin (Table 1).

### 2.2. Effect of Phellinus linteus Extract on Agonist-Induced Contraction in Mesenteric Arteries of Rats

**Figure 2:**
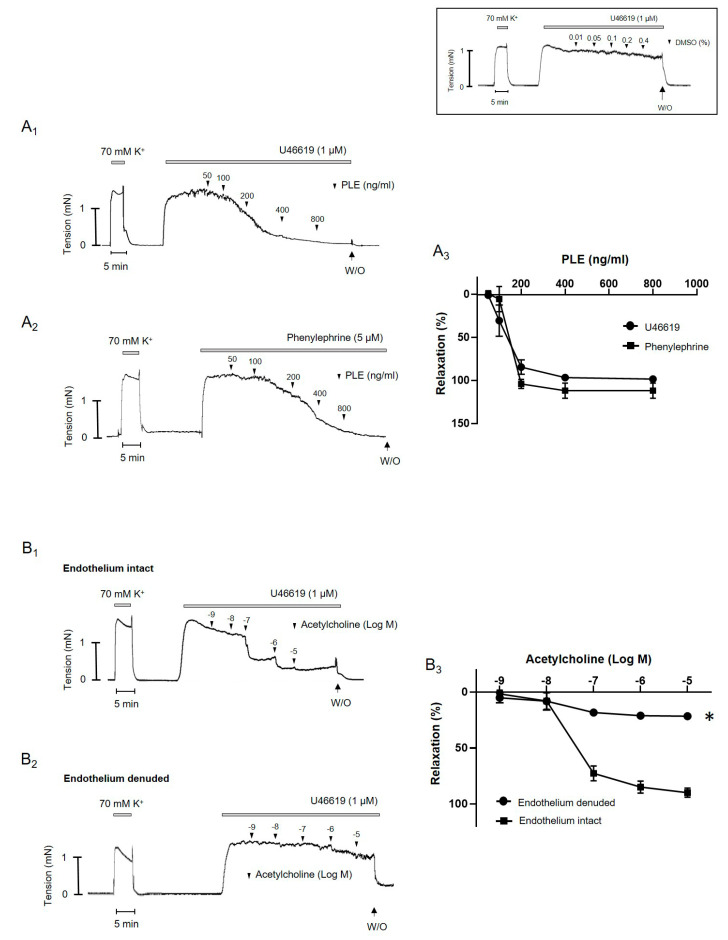
*Phellinus linteus* extract induces vasodilation in mesenteric arteries of rats. (**A_1_**–**A_3_**), data showing responses to cumulative administration of *Phellinus linteus* (50 ng/mL–800 ng/mL) on U46619 (**A_1_**) and phenylephrine (**A_2_**)-induced contraction. Statistical analysis of the relaxation response to *Phellinus linteus* (**A_3_**). (**B_1_**–**B_3_**), data showing responses to cumulative administration of acetylcholine (10^−9^ M–10^−5^ M) on U46619-induced contraction in endothelium intact (**B_1_**) and endothelium denuded (**B_2_**) mesenteric arteries. Statistical analysis of the relaxation response to acetylcholine (**B_3_**). Inset, representative trace showing responses to vehicle DMSO (0.01–0.4%). Mean ± SD (*n* = 5). * *p* < 0.05 for endothelium intact vs. endothelium denuded. (PLE: *Phellinus linteus* extract, W/O: wash out).

**Figure 3:**
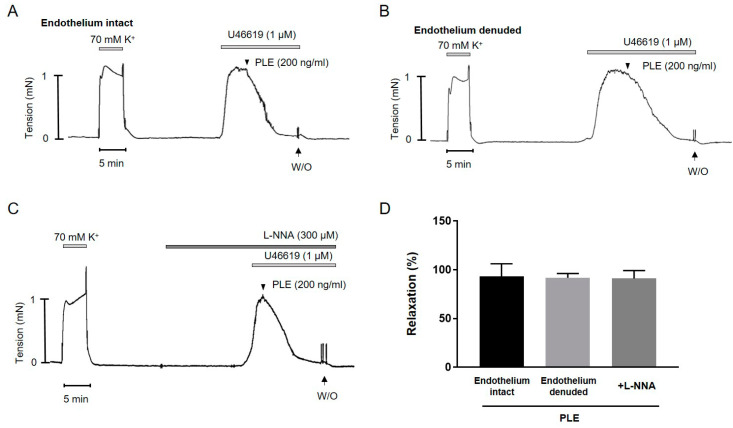
Involvement of endothelium in *Phellinus linteus* extract-induced relaxation. (**A**) Relaxation by *Phellinus linteus* extract in endothelium intact mesenteric artery pre-contracted with U46619 (1 μΜ). (**B**) Relaxation by *Phellinus linteus* extract in endothelium denuded mesenteric artery pre-contracted with U46619 (1 μΜ). (**C**) Relaxation by *Phellinus linteus* extract in mesenteric artery in the presence of l–NNA (300 μM). (**D**) Statistical analysis of the relaxation response of *Phellinus linteus* extract. Relaxation of arteries is expressed as the percentage of the contraction induced by U46619 (1 μΜ). Mean ± SD. (*n* = 5). (l–NNA: nomega–nitro–l–arginine).

*Phellinus linteus* extract induced relaxation in a dose-dependent manner in the rats’ mesenteric arteries pre-contracted with U46619 (1 μM) and phenylephrine (5 μM) (Figure 2(A_1_,A_2_)). There was no difference in vasodilatory effect of *Phellinus linteus* extract between U46619- and phenylephrine-induced contraction (Figure 2(A_3_)). The vehicle, dimethyl dulfoxide (DMSO, maximum of 0.4%) had no significant effect on the U46619-induced contraction (Figure 2 inset). To compare the effect of *Phellinus linteus* extract with another vasodilator, aceylcholine was administered in a U46619-induced contraction (Figure 3). Acetylcholine induced dose-dependent relaxation in a U46619-induced contraction in endothelium-intact mesenteric arteries (Figure 3(B_1_)), which was significantly abolished by endothelium removal (Figure 3(B_2_,B_3_)). These results suggested that *Phellinus linteus* extract can act as a vasodilator in the mesenteric arteries of rats.

### 2.3. Phellinus linteus Extract-Induced Endothelium-Independent Relaxation

To investigate the underlying mechanisms of *Phellinus linteus* extract-induced relaxation, *Phellinus linteus* extract was applied in endothelium-intact and endothelium-denuded mesenteric arteries (Figure 3A,B). There was no significant difference between endothelium-intact and endothelium-denuded mesenteric arteries. To confirm the effect of *Phellinus linteus* extract on the endothelium, the mesenteric arteries were pre-incubated with the endothelial nitric oxide synthase (eNOS) inhibitor nomega–nitro–l–arginine (l–NNA, 300 μM) for 20 min before being contracted with U46619 (1 μM, Figure 3C). The l–NNA did not affect the *Phellinus linteus* extract-induced relaxation, indicating that the relaxation effect of *Phellinus linteus* extract was not related to the endothelium. This result suggests that *Phellinus linteus* extract-induced relaxation is endothelium-independent.

### 2.4. Inhibition of Phellinus linteus Extract-Induced Relaxation by K^+^ Channel Blockers

**Figure 4:**
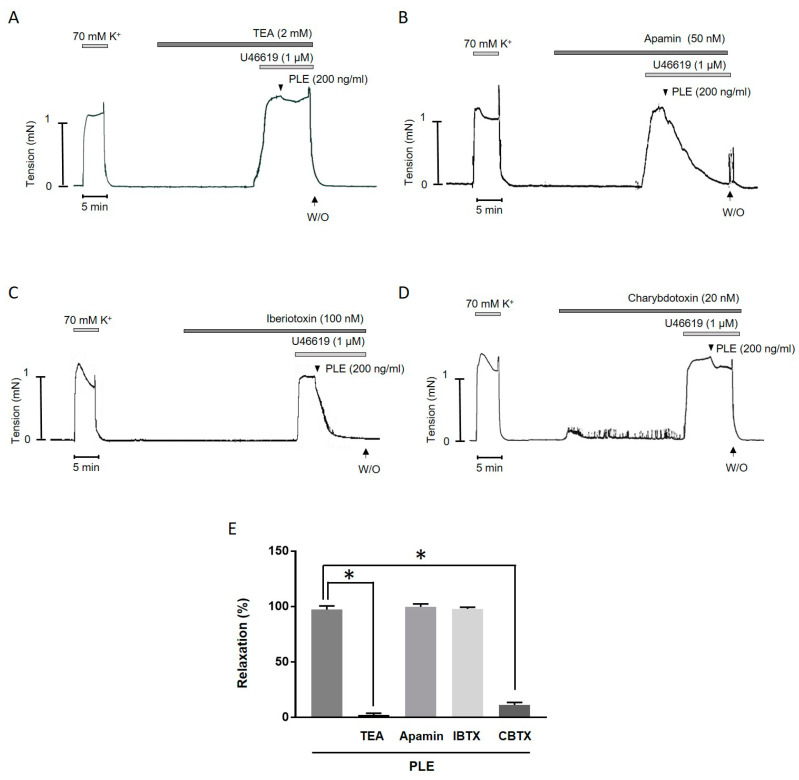
Involvement of K^+^ channel in *Phellinus linteus* extract-induced relaxation. (**A**) Effect of *Phellinus linteus* extract in the mesenteric artery pre-contracted with U46619 (1 μΜ) in the presence of TEA (2 mM). (**B**) Effect of *Phellinus linteus* extract in the mesenteric artery pre-contracted with U46619 (1 μΜ) in the presence of apamin (50 nM). (**C**) Effect of *Phellinus linteus* extract in the mesenteric artery pre-contracted with U46619 (1 μΜ) in the presence of iberiotoxin (100 nM). (**D**) Effect of *Phellinus linteus* extract in the mesenteric artery pre-contracted with U46619 (1 μΜ) in the presence of charybdotoxin (20 nM). (**E**) Statistical analysis of the relaxation response of *Phellinus linteus* extract in the presence of various blockers. Relaxation of arteries is expressed as the percentage of the contraction induced by U46619 (1 μΜ). Mean ± SD (*n* = 5). * *p* < 0.05 for control versus TEA or charybdotoxin. (TEA: tetraethylammonium, IBTX: iberiotoxin, CBTX: charybdotoxin).

To clarify the underlying mechanisms of the *Phellinus linteus* extract-induced relaxation, the mesenteric arteries were incubated with tetraethylammonium (TEA, 2 mM, Figure 4A), apamin (50 nM, Figure 4B), iberiotoxin (100 nM, Figure 4C), or charybdotoxin (20 nM, Figure 4D) for 20 min, and then *Phellinus linteus* extract was added. The non-selective K^+^ channel blocker, TEA, and the IK_ca_ blocker, charybdotoxin, significantly inhibited *Phellinus linteus* extract-induced relaxation, while the SK_ca_ channel blocker, the apamin, and the BK_ca_ channel blocker iberiotoxin did not affect the *Phellinus linteus* extract-induced vasodilation (Figure 4E). These results indicate that the IK_ca_ channel is involved in the relaxation induced by *Phellinus linteus* extract.

### 2.5. Effect of Phellinus linteus Extract on the Membrane Potential and Phosphorylation of 20 KDa Myosin Light Chain (MLC_20_)

**Figure 5:**
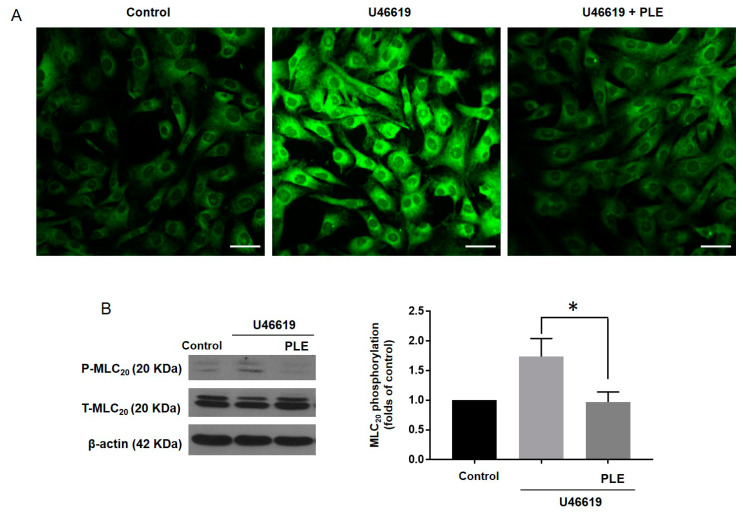
Effect of *Phellinus* linteus extract on the membrane potential and phosphorylation of 20 KDa myosin light chain (MLC_20_). (**A**) Representative images of fluorescence intensity of DiBAC_4_(3) in control VSMCs, VSMCs treated with U46619 (1 μΜ), and VSMCs co-treated with U46619 (1 μΜ) and *Phellinus linteus* extract (200 ng/mL) (scale bar: 50 μm). (**B**) Representative western blot analysis and quantitative data for phosphorylated MLC_20_ (P–MLC_20_) and total MLC_20_ (T–MLC_20_) in control VSMCs, VSMCs treated with U46619 (1 μΜ), and VSMCs co-treated with U46619 (1 μΜ) and *Phellinus linteus* extract (200 ng/mL). * *p* < 0.05 for VSMCs treated with U46619 vs. VSMCs co-treated with U46619 and *Phellinus linteus* extract. (*n* = 4).

To clarify whether *Phellinus linteus* extract-induced relaxation was produced by hyperpolarization in VSMCs, we measured the membrane potential using the voltage sensitive dye bis-(1,3-dibarbituric acid)-trimethine oxanol (DiBAC_4_(3)) and obtained confocal images. The application of U46619 (1 µM) increased the fluorescence intensity of the membrane potential in VSMCs compared to the control group. In the presence of *Phellinus linteus* extract (200 ng/mL), U46619 did not increase the fluorescence intensity of the membrane potential in the VSMCs (Figure 5A). To investigate whether the *Phellinus linteus* extract-induced relaxation was caused by the decreased phosphorylation of MLC_20_, we measured the phosphorylation and the expression level of MLC_20_ in the VSMCs (Figure 5). The administration of U46619 increased the phosphorylation level of MLC_20_ in the VSMCs compared to the control group. In the presence of *Phellinus linteus* extract, U46619 did not increase the phosphorylation level of MLC_20_ (Figure 5B). These results suggested that *Phellinus linteus* extract induced hyperpolarization and inhibited the subsequent phosphorylation of MLC_20_ in U46619-treated VSMCs.

## 3. Discussion

In this study, we investigated the vasodilatory effect of *Phellinus linteus* extract on the mesenteric arteries of rats. We found that this effect was independent of the endothelium. The mechanism of vasodilation induced by the *Phellinus linteus* extract involved the IK_Ca_ channel. The non-selective K^+^ channel blocker TEA and the specific IK_Ca_ channel blocker charybdotoxin inhibited the relaxation induced by the *Phellinus linteus* extract. We also found that the *Phellinus linteus* extract induced hyperpolarization of the VSMCs, which caused a decrease of the phosphorylated MLC_20_ and subsequent vasodilation of the mesenteric arteries.

In several experimental models, it has been reported that *Phellinus linteus* extract has several strong biological activities, such as anti-oxidative, immune-modulating, hypoglycemic, and hepatoprotective effect [18,19,20,21]. However, the vascular effect of *Phellinus linteus* extract has not been assessed. Thus, the present study is the first investigation to explore the vascular effect of this extract in resistance arteries. The vasodilatory effect of *Phellinus linteus* extract remained in endothelium-denuded mesenteric arteries of rats and in endothelium-intact arteries in the presence of l–NNA. Thus, the relaxation caused by the extract was independent of the endothelium. We found in the *Phellinus linteus* extract various compounds, including palmitic acid ethyl ester, linoleic acid, linoleic acid ethyl ester, lichesterol, 5,6-dihydroergosterol, 7-ergostenol, lupenone, and betulin. We assume that linoleic acid could play a critical role in the relaxation induced by *Phellinus linteus* extract since it has been reported that linoleic acid induced relaxation and hyperpolarization in the coronary arteries of pigs [22]. However, further studies are needed to clarify the exact compound that causes relaxation.

Vascular smooth muscle contraction is initiated by an elevation of intracellular Ca^2+^, which can result from either extracellular Ca^2+^ influx through calcium channels, or Ca^2+^ release from sarcoplasmic reticulum (SR). Extracellular Ca^2+^ influx via the voltage gated Ca^2+^ channel can be evoked by membrane depolarization. Inhibition of the K^+^ channels contributes to membrane depolarization. By contrast, activation of the K^+^ channels enables K^+^ efflux and leads to membrane hyperpolarization, which contributes to the closure of the voltage-dependent Ca^2+^ channels to block the influx of extracellular Ca^2+^, thereby inducing relaxation of the smooth muscle cells [23].

It is well known that at least four different types of K^+^ channels are expressed in VSMCs, including K_Ca_ channels, voltage-gated K^+^ (K_v_) channels, ATP-sensitive K^+^ (K_ATP_) channels, and inward-rectifier K^+^ channels [24]. K_Ca_ channels play a functional role by coupling the increase of intracellular Ca^2+^ to the hyperpolarization of the membrane potential. This feature enables K_Ca_ channels to play key roles in regulating cell excitability and K^+^ homeostasis [25]. The K_Ca_ channels are divided into three subfamilies that include small conductance K_Ca_ (SK_Ca_) channels, large or big K_Ca_ (BK_Ca_), and intermediate K_Ca_ (IK_Ca_). It has been reported that the IK_Ca_ channel seems to be involved in the VSMCs proliferation [26] and vasodilation of porcine coronary arteries [27].

In the present study, we found that *Phellinus linteus* extract-induced relaxation was abolished in the presence of the non-selective K^+^ channel inhibitor TEA. Thus, we assumed that the K^+^ channel is involved in the extract-induced vasodilation. To delineate which K^+^ channel is involved in the *Phellinus linteus* extract-induced relaxation, we used specific blockers of BK_ca_ (iberiotoxin), SK_ca_ (apamin), and IK_ca_ (charybdotoxin) channels. Interestingly, charybdotoxin significantly inhibited *Phellinus linteus* extract-induced relaxation, while apamin and iberiotoxin did not affect the extract-induced vasodilation. Charybdotoxin is known to block several K_Ca_ channels [28], however it is also known to specifically block the IK_ca_ channel in a concentration between 20 and 300 nM [29,30,31]. Additionally, in the present study we used a low dose (20 nM) of charybdotoxin to block only the IK_ca_ channel. Thus, we assume that the IK_ca_ channel could be involved in *Phellinus linteus* extract-induced vasodilation. To confirm the effect of the *Phellinus linteus* extract, we measured the membrane potential with the voltage-sensitive dye DiBAC_4_(3) in the primary isolated VSMCs. The membrane potential is one of the major contributors to the contractile activity of VSMCs. The activation of the IK_Ca_ channel and subsequent K^+^ efflux leads to membrane hyperpolarization, which causes vasodilation. We found that *Phellinus linteus* extract induced hyperpolarization of VSMCs. This result is consistent with the previous report that showed that *Phellinus linteus* extract induced hyperpolarization in a concentration-dependent manner in monocytes [15].

The increase of intracellular Ca^2+^ concentration activates Ca^2+^/calmodulin-dependent myosin light chain kinase (MLCK) and the phosphorylation of 20 KDa myosin light chain (MLC_20_) and thus induces smooth muscle contraction. The decrease of intracellular Ca^2+^ induces dephosphorylation of the MLC_20_ and thus smooth muscle relaxation [32,33]. Therefore, the phosphorylation of MLC_20_ is considered to be a key regulation step in smooth muscle contraction and relaxation. In the present study, we found that phosphorylated MLC_20_ is decreased in *Phellinus linteus* extract-treated VSMCs. These results indicate that the relaxation induced by *Phellinus linteus* extract involves hyperpolarization via IK_ca_ channel activation and subsequent decrease in MLC_20_ phosphorylation.

In conclusion, *Phellinus linteus* extract induces endothelium-independent relaxation in the mesenteric arteries of rats, and this effect involves the opening of IK_Ca_ channels, thereby hyperpolarizing the VSMCs. Thus, the IK_Ca_ channel may be a cellular target for the vasodilatory effects of *Phellinus linteus* extract in the vasculature.

## 4. Methods

All experiments were performed according to the Guide for the Care and Use of Laboratory Animals, published by the US National Institutes of Health (NIH publication No. 85-23, 2011) and were approved by the Ethics Committee and the Institutional Animal Care and Use Committee of Yonsei University College of Medicine (Approval number: 2019-0278).

### 4.1. Phellinus linteus Extract Preparation

Dried *Phellinus linteus* was purchased from the Gyeong-dong medicinal herb market (Seoul, Korea). The sample (1 kg) was ground into a powder and mixed in ethanol (5 L) by shaking for 24 h at 125 rpm (1.57× *g*). The ethanol extract was filtered through No. 42 filter paper (Whatman International Ltd., Middlesex, UK) with five replicates, and evaporated in a rotary evaporator (Eyela, Tokyo, Japan) under reduced pressure at 37 °C.

### 4.2. Gas Chromatography–Mass Spectrometry (GC/MS) Analysis

GC/MS analysis was performed by an Agilent 7890B gas chromatograph, equipped with a 5977A mass selective detector quadrupole mass spectrometer system (Palo Alto, CA, USA). The DB-5 MS capillary column (30 m × 0.25 mm i.d., 0.25 μm film thickness, 5% diphenyl-95% dimethylsiloxane phase) was obtained from J&W Scientific (Folsom, CA, USA). The GC oven temperature was maintained at 60 °C for 3 min, and then ramped to 320 °C at 10 °C per min. The sample was injected in the split mode, at a splitting ratio of 1:30. The temperatures of the GC injection port and MS interface were set at 300 °C. The mass selective detector was run in the electron impact (EI) mode, with an electron energy at 70 eV. The mass spectrometer was operated in the full scan mode between 40 and 600 amu. For the identification of the compounds, EI mass spectral library search (Wiley registry 7n edition, Wiley Science Solutions, Hoboken, NJ, USA) was used.

### 4.3. Tissue Preparation

In this experiment, 12-week-old male Sprague Dawley rats were used. The rats were sacrificed with isoflurane (5%), followed by CO_2_ inhalation. To confirm death, the rats were carefully checked for several signs, such as no response to toe pinch, no palpable heartbeat, and color change opacity in the eyes. After we confirmed death, the heart was excised immediately and then the mesenteric artery beds were removed and placed in ice-cold Krebs–Henseleit (K–H) solution (composition in mM: NaCl, 119; CaCl_2_, 2.5; NaHCO_3_, 25; MgSO_4_, 1.2; KH_2_PO_4_, 1.2; KCl, 4.6; and glucose, 11.1). Connective tissues and adipose tissues were removed under an optical microscope (model SZ-40, Olympus, Tokyo, Japan). The second or third branches of the mesenteric arteries (200–250 μm, inner diameter) were isolated and cut into 2–3 mm segments for subsequent analysis.

### 4.4. Isometric Tension Recording

The mesenteric artery segments were mounted in a myograph chamber (DMT, Arhaus, Denmark) for recording of isometric tension. Briefly, two steel wires (40 µm in diameter) were inserted into the lumen of the artery and then mounted according to the methods previously described [34]. After a 30-min equilibration period in a K–H solution bubbled with 5% CO_2_ + 95% O_2_ at 37 °C, the arteries were stretched to their optimal lumen diameter for active tension development. Vessel contractility was tested by exposure to a high K^+^ (70 mM) solution. Where required, the endothelium was mechanically denuded by rubbing the inner surface of an arterial segment with a wire. Removal of the endothelium was confirmed by the absence of relaxation from acetylcholine (10 µM) in the U46619 (thromboxane analogue, 1 µM) pre-contracted artery. After another wash step, the rings of the mesenteric arteries were pre-contracted with U46619 (1 µM), and at the steady maximal contraction, cumulative dose-response curves were obtained for the *Phellinus linteus* extract. To determine the involvement of the endothelium, the arteries were pre-incubated with l–NNA (300 µM), a nitric oxide synthase inhibitor, for 20 min before being contracted with U46619 (1 μM). To determine the effects of the K_ca_ channel blockers on vasodilation, the arterial segments were pre-treated with TEA (2 mM), iberiotoxin (100 nM), charybdotoxin (20 nM), or apamin (50 nM) for 20 min, then U46619 was administered.

### 4.5. Isolation and Culture of Vascular Smooth Muscle cells (VSMCs)

Vascular smooth muscle cells (VSMCs) were obtained as previously described [35]. Briefly, the aortas were excised, and fat and connective tissues were removed. And the lumen of the aorta was gently rubbed for removal of the endothelium. The aortas were cut into small segments and transferred into a tube containing elastase (0.5 mg/mL, Calbiochem, San Diego, CA, USA) and collagenase (1 mg/mL, Worthington Biomedical Corporation, Lakewood Township, NJ, USA) in Dulbecco’s Modified Eagle Medium (DMEM, Gibco, Waltham, MS, USA) at 37 °C for 30 min. After trituration and centrifugation, the cells were seeded in culture dishes (Corning, New York, NY, USA) and cultivated in DMEM supplemented with 10% fetal bovine serum (FBS, Gibco), 100 IU/mL penicillin (Gibco), and 100 μg/mL streptomycin (Gibco) at 37 °C, 5% CO_2_ with a humidified atmosphere. The early passage cells (between 2 and 4) were used.

### 4.6. Western Blot Analysis

The cultured VSMCs were frozen in liquid nitrogen after treatment with U46619, with or without *Phellinus linteus* extract. VSMCs were homogenized in an ice-cold lysis buffer, as described previously [36]. Western blot analysis was performed for total MLC_20_ and phosphorylated MLC_20_ (1:1000 dilution; Cell signaling, Boston, MA, USA). The blots were stripped and then reprobed with the β-actin antibody (1:2000 dilution; Santa Cruz Biotechnology, Santa Cruz, CA, USA) to verify equal loading between the samples.

### 4.7. Determination of Membrane Potential of Vascular Smooth Muscle Cells by Confocal Microscopy

The cells were seeded in a plate coated with poly–l–lysine for 24 h, then incubated with membrane potential-sensitive fluorescent dye DiBAC_4_(3) (5 μM) for 20 min. U46619 (1 μM) and *Phellinus linteus* extract (200 ng/mL) were added to the cells for 10 min, and the cells were washed with PBS 2–3 times. The cells were then fixed with formaldehyde (4%).

### 4.8. Drugs

The following drugs were used: U46619 (Tocris Bioscience, Ellisville, MO, USA), acetylcholine (Sigma-Aldrich, St Louis, MO, USA), DiBAC_4_(3) (Biotium, Fremont, CA, USA), and general laboratory reagents (Sigma-Aldrich, St Louis, MO, USA).

### 4.9. Statistical Analysis

Results were expressed as mean ± SD. One-way or two-way ANOVA was used to compare each parameter when appropriate. Comparisons between groups were performed with *t*-tests when the ANOVA test was statistically significant. Values of *p* < 0.05 were considered significant. Differences between specified groups were analyzed using the Student *t* test (2-tailed) for comparing two groups, with *p* < 0.05 considered statistically significant.

## Figures and Tables

**Table 1:** Bioactive compounds detected in *Phellinus linteus* extract. (PK: peak, RT: retention time).

PK	RT (min)	Tentative Compounds	Total (%)
1	19.68	palmitic acid ethyl ester	3.48
2	21.03	linoleic acid	10.39
3	21.27	linoleic acid ethyl ester	15.86
4	28.93	lichesterol	2.59
5	29.25	5,6-dihydroergosterol	3.26
6	29.69	7-ergostenol	2.80
7	30.40	lupenone	3.24
8	34.03	betulin	2.19

## References

[1] Tykocki N.R., Boerman E.M., Jackson W.F. (2017). Smooth Muscle Ion Channels and Regulation of Vascular Tone in Resistance Arteries and Arterioles. Compr. Physiol..

[2] Jaminon A., Reesink K.D., A Kroon A., Schurgers L.J. (2019). The Role of Vascular Smooth Muscle Cells in Arterial Remodeling: Focus on Calcification-Related Processes. Int. J. Mol. Sci..

[3] Brayden J.E. (1996). POTASSIUM CHANNELS IN VASCULAR SMOOTH MUSCLE. Clin. Exp. Pharmacol. Physiol..

[4] LeDoux J., Werner M.E., Brayden J.E., Nelson M.T. (2006). Calcium-Activated Potassium Channels and the Regulation of Vascular Tone. Physiology.

[5] Chen H., Tian T., Miao H., Zhao Y.-Y. (2016). Traditional uses, fermentation, phytochemistry and pharmacology of Phellinus linteus: A review. Fitoterapia.

[6] Hu T., Lin Q., Guo T., Yang T., Zhou W., Deng X., Yan J.-K., Luo Y., Ju M., Luo F. (2018). Polysaccharide isolated from Phellinus linteus mycelia exerts anti-inflammatory effects via MAPK and PPAR signaling pathways. Carbohydr. Polym..

[7] Kim B.-C., Jeon W.-K., Hong H.-Y., Jeon K.-B., Hahn J.-H., Kim Y.-M., Numazawa S., Yosida T., Park E.-H., Lim C.-J. (2007). The anti-inflammatory activity of Phellinus linteus (Berk. & M.A. Curt.) is mediated through the PKCδ/Nrf2/ARE signaling to up-regulation of heme oxygenase-1. J. Ethnopharmacol..

[8] Mei Y., Zhu H., Hu Q., Liu Y., Zhao S., Peng N., Liang Y. (2015). A novel polysaccharide from mycelia of cultured Phellinus linteus displays antitumor activity through apoptosis. Carbohydr. Polym..

[9] Chai Y., Wang G., Fan L., Zhao M. (2016). A proteomic analysis of mushroom polysaccharide-treated HepG2 cells. Sci. Rep..

[10] Wang H., Wu G., Park H.-J., Jiang P.P., Sit W.-H., Van Griensven L.J., Wan J.M. (2012). Protective effect of Phellinus linteus polysaccharide extracts against thioacetamide-induced liver fibrosis in rats: A proteomics analysis. Chin. Med..

[11] Huang S.C., Wang P.W., Kuo P.C., Hung H.Y., Pan T.L. (2018). Hepatoprotective Principles and Other Chemical Constituents from the Mycelium of Phellinus linteus. Molecules.

[12] Kim H.M., Kang J.S., Kim J.Y., Park S.-K., Kim H.S., Lee Y.J., Yun J., Hong J.T., Kim Y., Han S.-B. (2010). Evaluation of antidiabetic activity of polysaccharide isolated from Phellinus linteus in non-obese diabetic mouse. Int. Immunopharmacol..

[13] Park J.M., Lee J.S., Song J.E., Sim Y.C., Ha S.-J., Hong E.K. (2015). Cytoprotective Effect of Hispidin against Palmitate-Induced Lipotoxicity in C2C12 Myotubes. Molecules.

[14] Choi D.J., Cho S., Seo J.Y., Lee H.B., Park Y.I. (2016). Neuroprotective effects of the Phellinus linteus ethyl acetate extract against H2O2-induced apoptotic cell death of SK-N-MC cells. Nutr. Res..

[15] Van Griensven L.J.L.D., Verhoeven H.A. (2013). Phellinus linteus polysaccharide extracts increase the mitochondrial membrane potential and cause apoptotic death of THP-1 monocytes. Chin. Med..

[16] Lee Y.S., Kim Y.H., Shin E.K., Kim D.H., Lim S.S., Lee J.-Y., Kim J.-K. (2010). Anti-angiogenic activity of methanol extract of Phellinus linteus and its fractions. J. Ethnopharmacol..

[17] Song Y.S., Kim S.-H., Sa J.-H., Jin C., Lim C.-J., Park E.-H. (2003). Anti-angiogenic, antioxidant and xanthine oxidase inhibition activities of the mushroom Phellinus linteus. J. Ethnopharmacol..

[18] Shon Y.-H., Nam K.-S. (2003). Inhibition of cytochrome P450 isozymes in rat liver microsomes by polysaccharides derived from Phellinus linteus. Biotechnol. Lett..

[19] Fan G., Jian D., Sun M., Zhan Y., Sun F. (2015). Endogenous and Exogenous Calcium Involved in the Betulin Production from Submerged Culture of Phellinus linteus Induced by Hydrogen Sulfide. Appl. Biochem. Biotechnol..

[20] Nam S.W., Baek J.T., Kang S.B., Lee D.S., Kim J.I., Cho S.H., Park S.-H., Han J.-Y., Ahn B.M., Kim J.K. (2005). A case of the hepatocellular carcinoma during the pregnancy and metastasis to the left atrium. Korean J. Hepatol..

[21] Liu Y., Wang C., Li J., Mei Y., Liang Y. (2019). Hypoglycemic and Hypolipidemic Effects of Phellinus Linteus Mycelial Extract from Solid-State Culture in A Rat Model of Type 2 Diabetes. Nutrients.

[22] Pomposiello S.I., Alva M., Wilde D.W., Carretero O.A. (1998). Linoleic acid induces relaxation and hyperpolarization of the pig coronary artery. Hypertension.

[23] Jackson W.F. (2016). Potassium Channels in Regulation of Vascular Smooth Muscle Contraction and Growth. Adv. Pharmacol..

[24] Cheng J., Wen J., Wang N., Wang C., Xu Q., Yang Y. (2019). Ion Channels and Vascular Diseases. Arter. Thromb. Vasc. Boil..

[25] Kshatri A., González-Hernández A.J., Giraldez T. (2018). Physiological Roles and Therapeutic Potential of Ca2+ Activated Potassium Channels in the Nervous System. Front. Mol. Neurosci..

[26] Bi D., Toyama K., Lemaître V., Takai J., Fan F., Jenkins D.P., Wulff H., Gutterman D.D., Park F., Miura H. (2013). The Intermediate Conductance Calcium-activated Potassium Channel KCa3.1 Regulates Vascular Smooth Muscle Cell Proliferation via Controlling Calcium-dependent Signaling. J. Biol. Chem..

[27] Dalsgaard T., Kroigaard C., Misfeldt M., Bek T., Simonsen U. (2010). Openers of small conductance calcium-activated potassium channels selectively enhance NO-mediated bradykinin vasodilatation in porcine retinal arterioles. Br. J. Pharmacol..

[28] Hermann A., Erxleben C. (1987). Charybdotoxin selectively blocks small Ca-activated K channels in Aplysia neurons. J. Gen. Physiol..

[29] Sánchez-Carranza O., Torres-Rodríguez P., Darszon A., Treviño C.L., López-González I. (2015). Pharmacology of hSlo3 channels and their contribution in the capacitation-associated hyperpolarization of human sperm. Biochem. Biophys. Res. Commun..

[30] Logsdon N.J., Kang J., Togo J.A., Christian E.P., Aiyar J. (1997). A Novel Gene, hKCa4, Encodes the Calcium-activated Potassium Channel in Human T Lymphocytes. J. Boil. Chem..

[31] Anderson A.J., Harvey A.L., Rowan E.G., Strong P.N. (1988). Effects of charybdotoxin, a blocker of Ca2+ -activated K + channels, on motor nerve terminals. Br. J. Pharmacol..

[32] Ip K., Sobieszek A., Solomon D., Jiao Y., Paré P., Seow C. (2007). Physical Integrity of Smooth Muscle Myosin Filaments is Enhanced by Phosphorylation of the Regulatory Myosin Light Chain. Cell. Physiol. Biochem..

[33] Deng M., Ding W., Min X., Xia Y. (2010). MLCK-independent phosphorylation of MLC20 and its regulation by MAP kinase pathway in human bladder smooth muscle cells. Cytoskeleton.

[34] Sun J., Yang G.M., Tao T., Wei L.S., Pan Y., Zhu M.S. (2018). Isometric Contractility Measurement of the Mouse Mesenteric Artery Using Wire Myography. J. Vis. Exp..

[35] Choi S.-K., Kwon Y., Byeon S., Haam C.E., Lee Y.-H. (2020). AdipoRon, adiponectin receptor agonist, improves vascular function in the mesenteric arteries of type 2 diabetic mice. PLoS ONE.

[36] Choi S.-K., Ahn D.S., Lee Y.-H. (2008). Comparison of contractile mechanisms of sphingosylphosphorylcholine and sphingosine-1-phosphate in rabbit coronary artery. Cardiovasc. Res..

